# Postnatal growth and neurodevelopment at 2 years’ corrected age in extremely low birthweight infants

**DOI:** 10.1038/s41390-024-03054-1

**Published:** 2024-02-09

**Authors:** David A. Nyakotey, Angelica M. Clarke, Barbara E. Cormack, Frank H. Bloomfield, Jane E. Harding, on behalf of the ProVIDe Study Group, Frank H. Bloomfield, Yannan Jiang, Caroline A. Crowther, Barbara E. Cormack, Frank Bloomfield, Roland Broadbent, Frances McCaffrey, Adrienne Lynn, Carole Spencer, Nicola Ellis, Trish Graham, Michael Hewson, Harshad Patel, Mel Gibson, Natalie Wilkes, Arun Nair, Deborah Harris, Nicola Streifler, Stephanie Edwards, Rebecca Sisterson, Kimberly Akehurst, Mike Meyer, Aiza de Monteverde, Audrey Yu, Cristina Tapnio, Tanith Alexander, Barbara Cormack, Sabine Huth, Helen Liley, Suzanne Bates, Sue Jacobs, Brenda Argus, Emily Twitchell

**Affiliations:** 1 Liggins Institute, University of Auckland, Auckland, 1023 New Zealand; 2 Starship Child Health, Auckland City Hospital, Auckland, 1023 New Zealand; 3 Newborn Services, Auckland City Hospital, Auckland, New Zealand; 4 Department of Statistics, University of Auckland, Auckland, New Zealand; 5 Dunedin Hospital, Dunedin, New Zealand; 6 Christchurch Women’s Hospital, Christchurch, New Zealand; 7 Wellington Regional Hospital, Wellington, New Zealand; 8 Waikato Hospital, Hamilton, New Zealand; 9 Middlemore Hospital, Auckland, New Zealand; 10 Auckland City Hospital, Auckland, New Zealand; 11 Mater Health Services, Brisbane, QLD Australia; 12 The Royal Women’s Hospital, Melbourne, VIC Australia

## Abstract

**Background:**

Faltering postnatal growth in preterm babies is associated with adverse neurodevelopment. However, which growth reference is most helpful for predicting neurodevelopment is unknown. We examined associations between faltering growth and developmental delay in extremely low birthweight (ELBW) infants.

**Methods:**

We categorized faltering growth (z-score decrease ≥0.8 for weight/length, >1 for head circumference) between birth, 4 weeks, 36 weeks’ postmenstrual age and 2 years’ corrected age using fetal (Fenton, UK-WHO and Olsen) and healthy preterm (INTERGROWTH-21st) references. Associations between faltering growth and developmental delay were examined using binary logistic regression and area under the receiver operating curve (AUC).

**Results:**

In 327 infants, Olsen charts identified the highest prevalence of faltering growth (weight 37%, length 63%, head 45%). Agreement in classification was higher amongst fetal references (kappa coefficient, *ĸ* = 0.46–0.94) than between INTERGROWTH-21st and fetal references (*ĸ* = 0.10–0.81). Faltering growth in all measures between 4–36 weeks (odds ratio, OR 2.0–4.7) compared with other time intervals (OR 1.7–2.7) were more strongly associated with developmental delay, particularly motor delay (OR 2.0–4.7). All growth references were poorly predictive of developmental delay at 2 years (AUC ≤ 0.62).

**Conclusions:**

Faltering postnatal growth in ELBW infants is associated with, but is poorly predictive of, developmental delay at 2 years.

**Impact:**

- In babies born preterm, different growth references result in wide variation in categorization of faltering postnatal growth.
- Faltering growth in weight, length, and head circumference from 4 weeks to 36 weeks’ postmenstrual age are associated with developmental delay at 2 years’ corrected age, particularly motor delay.
- However, postnatal growth is a poor predictor of later developmental delay in extremely low birthweight infants irrespective of the growth reference used.

## Introduction

Survivors of preterm birth are at increased risk of long-term neurodevelopmental impairment,^1,2^ particularly those born at the earliest gestations.^3–5^ Later moderate to severe neurodevelopmental impairment is present in 32% of extremely low birthweight (ELBW) infants^2^ and is associated with poor postnatal growth during the neonatal period.^6^ Growth monitoring is recommended to detect faltering growth and allow appropriate intervention to optimize later development, but the most appropriate growth reference to do this is uncertain.

Widely used fetal growth charts such as Fenton,^7^ Olsen^8^ and UK-WHO,^9^ developed from cross-sectional birth data, do not appropriately represent intrauterine growth or postnatal growth of preterm infants,^10^ neither do they allow for factors contributing to preterm birth that have consequences for birthweight, or the differences between the intrauterine and neonatal intensive care (NICU) environment.^11^ Consequently, some experts recommend the use of INTERGROWTH- 21st Very Preterm Size at Birth References^12^ and INTERGROWTH- 21st Preterm Postnatal Growth standards,^13^ which were developed from longitudinal measures of normally- growing fetuses and infants subsequently born to healthy low risk mothers,^14^ for monitoring postnatal growth of preterm infants. However, owing to the very small numbers of infants born ≤33 weeks’ gestation included in INTERGROWTH- 21st, there are concerns that the INTERGROWTH- 21st charts are not appropriate for monitoring the postnatal growth of infants below this gestation.^15,16^

We examined the associations between postnatal growth faltering using four growth references (Fenton, UK-WHO, Olsen and INTERGROWTH-21st) and neurodevelopment at 2 years’ corrected age (CA) in infants born ELBW to determine how well-faltering growth using each of these references predicted developmental delay at 2 years’ CA, and the period of faltering growth that was the best predictor.

## Methods

### Study design and population

We undertook a secondary analysis of data from the Protein Intravenous Nutrition on Development (ProVIDe) trial, a multicenter (New Zealand and Australia), double-blinded, randomized controlled trial which recruited 434 ELBW babies from 2014 to 2018.^17^ Infants were eligible if birthweight was <1000 g and an umbilical arterial catheter (UAC) had been inserted. Infants admitted to NICU >24 h after birth, multiple birth >2 infants, genetic and other known abnormality, a congenital disorder impairing growth or in danger of imminent death were excluded. Participants were randomized to receive 1 g/d amino acid solution or placebo (saline) via the UAC for the first 5 postnatal days in addition to standard intravenous nutrition. The primary outcome was survival free of neurodevelopmental disability at 2 years’ CA. The ProVIDe trial was registered (Australian New Zealand Clinical Trials Registry: ACTRN12612001084875) and approved by the Northern B Health and Disability Committee (No 13/NTB/84) and the Children’s Health Queensland Hospital and Health Service Human Research Ethics Committee (HREC/16/QRCH/224) in New Zealand and Australia respectively. Parents or caregivers of each participant provided written informed consent.

For this study, we included all ProVIDe trial participants who underwent neurodevelopmental assessment at 2 years’ CA.

### Anthropometry

Head circumference, weight and height were measured at birth, 4 weeks’ and 36 weeks’ postmenstrual age (PMA) and 2 years’ CA. Head circumference was measured with a non-distensible tape measure. From birth until discharge, babies were weighed naked to the nearest 10 g using digital scales and their crown-to-heel length was measured by trained staff to the nearest 1 mm using a Harpenden or similar neonatometer (Holtain Ltd, Dyfed, Wales). At 2 years, children were weighed, without footwear and in minimal clothing, on a digital scale to the nearest 100 g. Their height was measured with a wall-mounted stadiometer to the nearest 1 mm.

### Neurodevelopmental outcomes assessment

Cognitive, language and motor skills were assessed using Bayley Scales of Infant and Toddler Development Edition 3 (Bayley-III)^18^ at 2 years’ CA. The assessment also included a neurological examination for the diagnosis of cerebral palsy (loss of motor function and abnormalities of muscle tone and power), deafness (required use of hearing aid(s) or worse) and blindness (visual acuity in both eyes worse than 6/60). Severity of cerebral palsy was classified using the Gross Motor Function Classification System (GMFCS).^19^

A child was classified as having neurodisability if they had at least one of: cerebral palsy; deafness; blindness, or developmental delay (defined as a standardized score of <−1 SD on any of the Bayley- III cognitive, language or motor composite scores). The severity of neurodisability was categorized as:^20^

#### Mild

Mild cerebral palsy (walking at 2 years with only minimal movement limitations; GMFCS level 1), or suspected developmental delay (standardized score −2 SD to <−1  SD on any one of Bayley- III motor, cognitive or language composite scores)

#### Moderate

Moderate cerebral palsy (non-ambulant at 2 years but likely to ambulate later; GMFCS level 2 or 3), or deafness, or moderate developmental delay (standardized score from −3 SD to <−2 SD on any one of the Bayley- III composite scores)

#### Severe

Severe cerebral palsy (non-ambulant; GMFCS level 4 or 5), severe developmental delay (standardized score < 3 SD on any one of the Bayley- III composite scores) or blindness.

### Statistical analyses

We calculated weight, length/ height and head circumference *z*- scores at birth, 4 weeks’ and 36 weeks’ PMA using the four growth charts (Fenton, UK-WHO, Olsen and INTERGROWTH-21st). For INTERGROWTH-21st, we used the Very Preterm Size at Birth References at birth and the Preterm Postnatal Growth standards at other time points. At 2 years’ CA, we used the WHO Child Growth Standards. The INTERGROWTH-21st Very Preterm Size at Birth reference begins at 24 weeks. Therefore, for babies born at 23 weeks’ gestation, birth *z*-scores using INTERGROWTH-21st were extrapolated as follows. The reference *z*-score for weight, length and head circumference were extracted separately for boys and girls from the INTERGROWTH-21st website.^21^ Each outcome (weight, length, height) then was plotted as a function of gestational age and the likely function determined. For length and head circumference (linear relation), ordinary least squares regression was used to estimate the slope and intercept for each *z*-score and then extrapolated for lower gestational ages. The same approach was used for birthweight (exponential relation) using nonlinear regression to fit the exponential function Vo*exp (K*gestational age) to each of the *z*-score data tables. The goodness of each model fit was verified by inspection of residuals. The UK-WHO length *z*-scores were not available below 27 weeks’ gestation and were treated as missing data not extrapolated because this was unlikely to be accurate over the missing 4 weeks.

Faltering growth was defined as follows: faltering weight gain as weight *z*-score decline ≥0.8, further classified as mild (0.8–1.2), moderate (>1.2–2) or severe (≥2);^10,22^ faltering linear growth as length *z*-score decline ≥0.8,^10^ and faltering head growth as head circumference *z*- score decline >1.^23^

We compared the prevalence of neurodisability between infants with and without faltering growth using Chi-square and Fisher’s exact tests. We examined the association between growth (change in *z*-scores over time) and Bayley III composite scores using Pearson’s correlations. We estimated the odds of developmental delay at 2 years’ CA among infants with and without faltering growth using binary logistic regression. We examined faltering growth as a predictor of developmental delay by calculating sensitivity, specificity and area under the receiver operating characteristic curve (AUC).

We used IBM SPSS Statistics version 28 and SAS (version 9.4 SAS Institute Inc, Cary NC) for statistical analyses. Continuous variables are presented as means with standard deviations (SD) or median and interquartile range and categorical variables as frequencies and percentages. We measured inter-observer agreement of faltering growth (categorical classification) between the growth references using Cohen’s kappa coefficient with 95% confidence intervals (CI). We undertook a sensitivity analyses excluding infants born before 24 weeks’ whose *z*-scores were calculated by extrapolation, and excluding small-for-gestational-age (SGA) infants (birthweight <10th centile using each of the four growth charts). A *p* value ≤ 0.05 was considered statistically significant for all analyses.

## Results

**Fig. 1:**
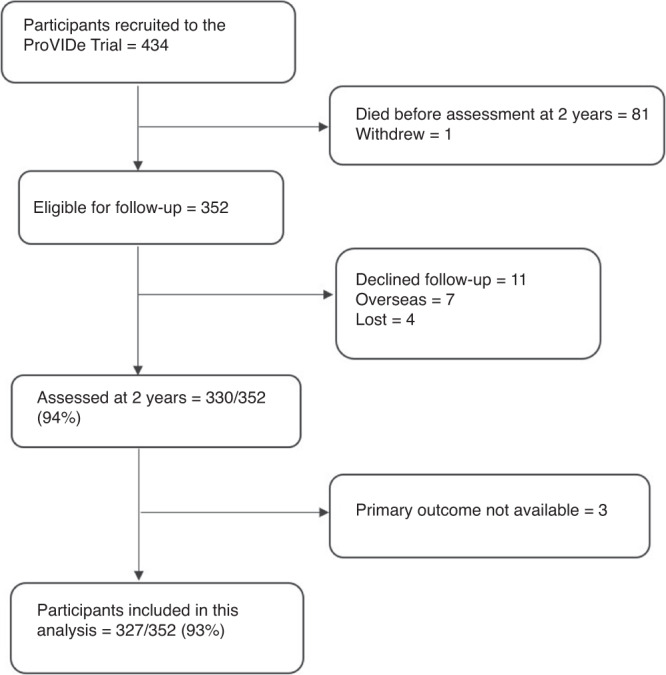
Flowchart for participants included in this study. Data presented as *n* (%). ProVIDe Protein Intravenous Nutrition on Development.

Of the 434 infants recruited to the ProVIDe trial, 352 were eligible for assessment at 2 years’ CA and 330/352 (94%) were assessed. Our study included 327/352 (93%) participants who had data available for the primary outcome of neurodisability (Fig. 1). The median gestational age was 26 weeks and median birthweight was 797 g (Table 1). European was the most common ethnicity (49%) and almost a third of mothers (33%) had a university degree. At 2 years’ CA, neurodisability was present in 128/327 (39%), with language the most common domain affected (32%).

**Table 1:** Sociodemographic and clinical characteristics of infants and their mothers.

Characteristic	Median (range) or *n* (%)
Infants at birth, *n* = 327	
Gestational age (weeks)	26 (23–31)
Weight (g)	797 (416–998)
Length (cm)	33.2 (27.0–39.0)
Head circumference (cm)	23.5 (19.5–27.0)
Singleton	259 (79.2)
Female	177 (54.1)
Infants at 2 years, *n* = 327	
Neurodisability	128 (39.1)
^ a^Cognition score <85	51 (15.9)
^ a^Language score <85	101 (31.8)
^ a^Motor score <85	53 (16.6)
Cerebral palsy	22 (6.8)
Deafness	8 (2.5)
Blindness	2 (0.6)
^ a^Cognition score	95 (55–145)
^ a^Language score	91 (47–153)
^ a^Motor score	97 (46–139)
^b^Mothers, *n* = 310	
Ethnicity	
European	151 (48.7)
Māori	71 (22.9)
Pacific Islander	26 (8.4)
Asian	56 (18.1)
Other	6 (1.9)
University degree	101 (32.6)

^a^Bayley III composite scores.

^b^Some mothers had only one twin recruited to the ProVIDe trial.

The proportions of participants classified as having faltering growth were different between growth references at all time periods (Table 2). Similarly, mean z-scores and mean change in *z*-scores were significantly different (Supplementary Table 1). The highest prevalence of faltering growth from birth to 4 weeks and from birth to 36 weeks’ PMA was recorded using the Olsen growth reference. The INTERGROWTH- 21st growth standard classified more participants as having faltering growth from 4 weeks to 36 weeks’ PMA and from 36 weeks’ PMA to 2 years.

**Table 2:** Prevalence of faltering growth at various time intervals using Fenton 2013, UK-WHO, Olsen 2010, and INTERGROWTH-21st.

	Fenton	UK-WHO	Olsen	IG-21st	*p* value
Birth to 4 weeks					
Faltering weight gain (any)	99 (30.3)	83 (25.4)	120 (36.7)	13 (4.0)	<0.001
Mild	60 (18.3)	47 (14.4)	59 (18.0)	12 (3.7)	
Moderate	38 (11.6)	34 (10.4)	51 (15.6)	1 (0.3)	
Severe	1 (0.3)	2 (0.6)	3 (3.1)	0 (0.0)	
Faltering linear growth	153 (49.4)	58 (25.1)	158 (51.0)	46 (14.8)	<0.001
Faltering head growth	190 (58.5)	179 (55.4)	193 (59.8)	171 (52.9)	0.001
Birth to 36 weeks					
Faltering weight gain (any)	114 (35.2)	72 (22.2)	105 (32.4)	38 (11.7)	<0.001
Mild	47 (14.5)	35 (10.8)	38 (11.7)	17 (5.2)	
Moderate	50 (15.4)	26 (8.0)	52 (16.0)	17 (5.2)	
Severe	17 (5.2)	11 (3.4)	15 (4.6)	4 (1.2)	
Faltering linear growth	184 (60.1)	81 (35.8)	193 (63.1)	136 (44.4)	<0.001
Faltering head growth	138 (43.0)	99 (30.8)	145 (45.2)	123 (38.3)	<0.001
4 weeks to 36 weeks					
Faltering weight gain (any)	47 (14.5)	29 (9.0)	34 (10.5)	83 (25.6)	<0.001
Mild	28 (8.6)	11 (3.4)	16 (4.9)	33 (10.2)	
Moderate	17 (5.2)	17 (5.2)	17 (5.3)	38 (11.7)	
Severe	2 (0.6)	1 (0.3)	1 (0.3)	12 (3.7)	
Faltering linear growth	86 (27.9)	66 (21.4)	83 (26.9)	139 (45.1)	<0.001
Faltering head growth	18 (5.7)	15 (4.7)	18 (5.7)	37 (11.7)	<0.001
36 weeks to 2 years					
Faltering weight gain (any)	42 (13.3)	52 (16.5)	45 (14.2)	97 (30.7)	<0.001
Mild	19 (6.0)	20 (6.3)	19 (6.0)	35 (11.1)	
Moderate	20 (6.3)	28 (8.9)	23 (7.3)	44 (13.9)	
Severe	3 (0.9)	4 (1.3)	3 (0.9)	18 (5.7)	
Faltering linear growth	23 (7.7)	24 (8.0)	21 (7.0)	39 (13.0)	<0.001
Faltering head growth	16 (5.4)	18 (6.0)	13 (4.3)	21 (7.0)	0.007

Data presented as *n* (%). IG-21st = INTERGROWTH-21st. Faltering head growth = decline in head circumference *z*-scores > 1. Faltering linear growth = decline in length *z*-scores ≥ 0.8. Faltering weight gain (any) = decline in weight *z*-scores ≥ 0.8; mild = decline in weight *z*-scores 0.8–1.2; moderate = decline in weight *z*-scores >1.2–2; severe = decline in weight *z*-scores > 2; Cochran’s Q test was used to compare differences in the proportion of faltering growth between growth references.

The proportion of agreement (kappa coefficient) in the classification of faltering growth was higher (moderate to excellent) among the three fetal references (Fenton 2013, Olsen 2010 and UK-WHO) than between the INTERGROWTH- 21st healthy preterm growth standard and the fetal references (poor to good) for each period of growth assessed (Table 3). The proportion of agreement between INTERGROWTH- 21st and the fetal references was lowest for faltering weight gain and highest for faltering head growth.

**Table 3:** Agreement in classification of faltering growth between growth charts.

	Faltering weight gain (any) *ĸ* (95% CI)			Faltering linear growth *ĸ* (95% CI)			Faltering head growth *ĸ* (95% CI)		
	UK-WHO	Olsen	IG-21st	UK-WHO	Olsen	IG-21st	UK-WHO	Olsen	IG-21st
Birth to 4 weeks									
Fenton	0.82 (0.75–0.89)	0.82 (0.75–0.88)	0.14 (0.05–0.22)	0.54 (0.43–0.64)	0.85 (0.79–0.91)	0.28 (0.20–0.36)	0.88 (0.83–0.93)	0.89 (0.84–0.94)	0.68 (0.60–0.76)
UK-WHO	-	0.74 (0.66–0.82)	0.17 (0.07–0.27)	-	0.48 (0.38–0.59)	0.50 (0.38–0.64)	-	0.87 (0.82–0.93)	0.59 (0.50–0.68)
Olsen	-	-	0.10 (0.04–0.17)	-	-	0.20 (0.12–0.28)	-	-	0.62 (0.54–0.71)
Birth to 36 weeks									
Fenton	0.69 (0.61–0.77)	0.73 (0.65–0.81)	0.39 (0.30–0.49)	0.55 (0.45–0.65)	0.81 (0.75–0.88)	0.64 (0.56–0.72)	0.73 (0.65–0.81)	0.78 (0.71–0.85)	0.70 (0.62–0.78)
UK-WHO	-	0.72 (0.63–0.8)	0.64 (0.53–0.75)	-	0.46 (0.36–0.57)	0.75 (0.65–0.84)	-	0.66 (0.58–0.75)	0.70 (0.62–0.78)
Olsen	-	-	0.40 (0.3–0.50)	-	-	0.51 (0.42–0.60)	-	-	0.57 (0.48–0.66)
4 weeks to 36 weeks									
Fenton	0.70 (0.58–0.83)	0.62 (0.49–0.75)	0.62 (0.52–0.73)	0.72 (0.63–0.81)	0.81 (0.74–0.89)	0.64 (0.56–0.72)	0.90 (0.79–1.01)	0.71 (0.53–0.88)	0.59 (0.43–0.75)
UK-WHO	-	0.81 (0.7–0.92)	0.42 (0.31–0.54)	-	0.73 (0.64–0.82)	0.50 (0.41–0.59)	-	0.71 (0.53–0.89)	0.51 (0.34–0.68)
Olsen	-	-	0.49 (0.37–0.60)	-	-	0.61 (0.52–0.69)	-	-	0.47 (0.30–0.64)
36 weeks to 2 years									
Fenton	0.88 (0.8–0.95)	0.88 (0.8–0.96)	0.51 (0.41–0.62)	0.84 (0.72–0.96)	0.9 (0.8–1)	0.68 (0.54–0.82)	0.94 (0.85–1.03)	0.89 (0.77–1.02)	0.80 (0.65–0.95)
UK-WHO	-	0.89 (0.82–0.96)	0.62 (0.52–0.71)	-	0.78 (0.64–0.92)	0.70 (0.56–0.84)	-	0.83 (0.68–0.98)	0.81 (0.67– 0.95)
Olsen	-	-	0.55 (0.44–0.65)	-	-	0.60 (0.44–0.75)	-	-	0.69 (0.51–0.87)

Faltering weight gain = decline in weight z-scores ≥ 0.8. Faltering linear growth = decline in length z-scores ≥ 0.8. Faltering head growth = decline in head circumference z-scores > 1.

*IG-21st* INTERGROWTH-21st, *ĸ* kappa coefficient, *95% CI* confidence interval.

The prevalence of any neurodisability was 37% higher in participants with faltering head growth than in participants with adequate head growth from birth to 4 weeks using both Fenton (*p* = 0.031) and INTERGROWTH-21st (*p* = 0.026) (Table 4). From birth to 36 weeks’ PMA, participants classified by INTERGROWTH-21st, but not the fetal references, as having faltering length and head growth had 55% (*p* = 0.002) and 42% (*p* = 0.012) higher prevalence of any neurodisability compared to those who had adequate length and head growth. Similarly, participants classified as having faltering growth in length from 4 weeks to 36 weeks by INTERGROWTH-21st had 46% (*p* = 0.008) higher prevalence of any neurodisability compared to those who had adequate length growth, whereas the differences in prevalence were smaller (0−26% for length and 18−25% for head growth) and not statistically significant for the fetal references. Generally, these increased strengths of associations were seen for both mild and moderate/severe disability. In contrast, the prevalence of any neurodisability was not significantly increased for infants who had any faltering weight gain. However, moderate/ severe faltering weight gain was associated with 19% to 75% higher incidence of moderate/ severe neurodisability compared to mild faltering weight gain.

**Table 4:** Prevalence of neurodisability at 2 years’ corrected age in infants with and without faltering growth, classified using Fenton 2013, UK-WHO, Olsen 2010, and INTERGROWTH-21st.

	Fenton			UK-WHO			Olsen			IG-21st		
Neurodisability	Any	Mild	Moderate/severe	Any	Mild	Moderate/severe	Any	Mild	Moderate/severe	Any	Mild	Moderate/severe
Birth to 4 weeks												
*Weight*												
Adequate Growth	82/228 (36.0)	52/227 (22.9)	29/227 (12.8)	93/244 (38.1)	61/243 (25.1)	31/243 (12.8)	76/207 (36.7)	48/207 (23.2)	28/207 (13.5)	124/314 (39.5)	83/313 (26.5)	40/313 (12.8)
Any Faltering Growth	46/99 (46.5)	34/99 (34.3)	12/99 (12.1)	35/83 (42.2)	25/83 (30.1)	10/83 (12.0)	52/120 (43.3)	38/119 (31.9)	13/119 (10.9)	4/13 (30.8)	3/13 (23.1)	1/13 (7.7)
* p* value	*0.074*	*0.093*		*0.513*	*0.669*		*0.237*	*0.216*		*0.773*	*0.794*	
Mild	28/60 (46.7)	22/60 (36.7)	6/60 (10.0)	19/47 (40.4)	13/47 (27.7)	6/47 (12.8)	28/59 (47.5)	21/58 (36.2)	6/58 (10.3)	4/12 (33.3)	3/12 (25.0)	1/12 (8.3)
Moderate/severe	18/39 (46.2)	12/39 (30.8)	6/39 (15.4)	16/36 (44.4)	12/36 (33.3)	4/36 (11.1)	24/61 (39.3)	17/61 (27.9)	7/61 (11.5)	0/1 (0.0)	0/0 (0.0)	0/0 (0.0)
* p* value	*0.202*	*0.230*		*0.754*	*0.886*		*0.329*	*0.389*		*0.661*	*0.925*	
*Length*												
Adequate Growth	57/157 (36.3)	37/156 (23.7)	19/156 (12.2)	62/173 (35.8)	42/172 (24.4)	19/172 (11.0)	56/152 (36.8)	37/151 (24.5)	18/151 (11.9)	101/264 (38.3)	69/263 (26.2)	31/263 (11.8)
Faltering Growth	64/153 (41.8)	46/153 (30.1)	18/153 (11.8)	20/58 (34.5)	19/58 (32.8)	1/58 (1.7)	65/158 (41.1)	46/158 (29.1)	19/158 (12.0)	20/46 (43.5)	14/46 (30.4)	6/46 (13.0)
* p* value	*0.319*	*0.446*		*0.852*	*0.065*		*0.438*	*0.640*		*0.503*	*0.779*	
*Head circumference*												
Adequate Growth	43/133 (32.3)	25/133 (18.8)	18/133 (13.5)	49/144 (34.0)	30/144 (20.8)	19/144 (13.2)	48/130 (36.9)	28/130 (21.5)	20/130 (15.4)	50/152 (32.9)	26/152 (17.1)	24/152 (15.8)
Faltering Growth	84/190 (44.2)	60/189 (31.7)	23/189 (12.2)	78/179 (43.6)	55/178 (30.9)	22/178 (12.4)	79/193 (40.9)	57/192 (29.7)	21/192 (10.9)	77/171 (45.0)	59/170 (34.7)	17/170 (10.0)
* p* value	**0.031**	**0.033**		*0.081*	*0.122*		*0.469*	*0.189*		**0.026**	**0.001**	
Birth to 36 weeks												
*Weight*												
Adequate Growth	87/210 (41.4)	63/210 (30.0)	24/210 (11.4)	102/252 (40.5)	75/252 (29.8)	27/252 (10.7)	86/219 (39.3)	62/219 (28.3)	24/219 (11.0)	113/286 (39.5)	80/286 (28.0)	33/286 (11.5)
Any Faltering Growth	41/114 (36.0)	23/113 (20.4)	17/113 (15.0)	26/72 (36.1)	11/71 (15.5)	14/71 (19.7)	42/105 (40.0)	24/104 (23.1)	17/104 (16.3)	15/38 (39.5)	6/37 (16.2)	8/37 (21.6)
* p* value	*0.337*	*0.152*		*0.504*	**0.018**		*0.900*	*0.311*		*0.997*	*0.114*	
Mild	14/47 (29.8)	11/47 (23.4)	3/47 (6.4)	12/35 (34.3)	7/35 (20.0)	5/35 (14.3)	19/38 (50.0)	13/38 (34.2)	6/38 (15.8)	9/17 (52.9)	4/16 (25.0)	4/16 (25.0)
Moderate/severe	27/67 (40.3)	12/66 (18.2)	14/66 (21.2)	14/37 (37.8)	4/36 (11.1)	9/36 (25.0)	23/67 (34.3)	11/66 (16.7)	11/66 (16.7)	6/21 (28.6)	2/21 (9.5)	4/21 (19.0)
* p* value	*0.333*	*0.056*		*0.763*	**0.038**		*0.285*	*0.182*		*0.311*	*0.192*	
*Length*												
Adequate Growth	41/122 (33.6)	28/122 (23.0)	13/122 (10.7)	51/145 (35.2)	36/145 (24.8)	15/145 (10.3)	38/113 (33.6)	29/113 (25.7)	9/113 (8.0)	53/170 (31.2)	36/170 (21.2)	17/170 (10.0)
Faltering Growth	78/184 (42.4)	53/183 (29.0)	24/183 (13.1)	28/81 (34.6)	22/80 (27.5)	5/80 (6.3)	81/193 (42.0)	52/192 (27.1)	28/192 (14.6)	66/136 (48.5)	45/135 (33.3)	20/135 (14.8)
* p* value	*0.123*	*0.330*		*0.927*	*0.568*		*0.149*	*0.185*		**0.002**	**0.010**	
*Head circumference*												
Adequate Growth	65/183 (35.5)	45/182 (24.7)	19/182 (10.4)	82/222 (36.9)	60/221 (27.1)	21/221 (9.5)	64/176 (36.4)	45/175 (25.7)	18/175 (10.3)	67/198 (33.8)	49/197 (24.9)	17/197 (8.6)
Faltering Growth	61/138 (44.2)	39/138 (28.3)	22/138 (15.9)	44/99 (44.4)	24/99 (24.2)	20/99 (20.2)	62/145 (42.8)	39/145 (26.9)	23/145 (15.9)	59/123 (48.0)	35/123 (28.5)	24/123 (19.5)
* p* value	*0.115*	*0.194*		*0.203*	**0.030**		*0.243*	*0.278*		**0.012**	**0.007**	
4 weeks to 36 weeks												
*Weight*												
Adequate Growth	112/277 (40.4)	82/277 (29.6)	30/277 (10.8)	119/295 (40.3)	83/294 (28.2)	35/294 (11.9)	116/290 (40.0)	81/289 (28.0)	34/289 (11.8)	90/241 (37.3)	68/241 (28.2)	22/241 (9.1)
Any Faltering Growth	16/47 (34.0)	4/46 (8.7)	11/46 (23.9)	9/29 (31.0)	3/29 (10.3)	6/29 (20.7)	12/34 (35.3)	5/34 (14.7)	7/34 (20.6)	38/83 (45.8)	18/82 (22.0)	19/82 (23.2)
* p* value	*0.407*	**0.002**		*0.328*	*0.077*		*0.595*	*0.136*		*0.175*	**0.004**	
Mild	9/28 (32.1)	2/27 (7.4)	6/27 (22.2)	3/11 (27.3)	2/11 (18.2)	1/11 (9.1)	6/16 (37.5)	4/16 (25.0)	2/16 (12.5)	18/33 (54.5)	11/32 (34.4)	6/32 (18.8)
Moderate/severe	7/19 (36.8)	2/19 (10.5)	5/19 (26.3)	6/18 (33.3)	1/18 (5.6)	5/18 (27.8)	6/18 (33.3)	1/18 (5.6)	5/18 (27.8)	20/50 (40.0)	7/50 (14.0)	13/50 (26.0)
* p* value	*0.673*	**0.014**		*0.588*	*0.113*		*0.842*	*0.148*		*0.165*	**0.004**	
*Length*												
Adequate Growth	81/222 (36.5)	57/222 (25.7)	24/222 (10.8)	95/242 (39.3)	67/242 (27.7)	28/242 (11.6)	84/225 (37.3)	60/225 (26.7)	24/225 (10.7)	55/169 (32.5)	41/169 (24.3)	14/169 (8.3)
Faltering Growth	40/86 (46.4)	25/85 (29.4)	14/85 (16.5)	26/66 (39.4)	15/65 (23.1)	10/65 (15.4)	37/83 (44.6)	22/82 (26.8)	14/82 (17.1)	66/139 (47.5)	41/138 (29.7)	24/138 (17.4)
* p* value	*0.106*	*0.246*		*0.984*	*0.602*		*0.248*	*0.298*		**0.008**	**0.014**	
*Head circumference*												
Adequate Growth	114/299 (38.1)	78/298 (26.2)	35/298 (11.7)	116/302 (38.4)	79/301 (26.2)	36/301 (12.0)	114/299 (38.1)	77/298 (25.8)	36/298 (12.1)	105/280 (37.5)	76/279 (27.2)	28/279 (10.0)
Faltering Growth	11/18 (61.1)	5/18 (27.8)	6/18 (33.3)	9/15 (60.0)	4/15 (26.7)	5/15 (33.3)	11/18 (61.1)	6/18 (33.3)	5/18 (27.8)	20/37 (54.1)	7/37 (18.9)	13/37 (35.1)
* p* value	*0.053*	**0.022**		*0.095*	**0.046**		*0.053*	*0.079*		*0.053*	**<0.001**	
36 weeks to 2 years												
*Weight*												
Adequate Growth	110/274 (40.1)	72/273 (26.4)	37/273 (13.6)	106/264 (40.2)	70/263 (26.6)	35/263 (13.3)	107/271 (39.5)	69/270 (25.6)	37/270 (13.7)	92/219 (42.0)	59/218 (27.1)	32/218 (14.7)
Any Faltering Growth	16/342 (38.1)	12/42 (28.6)	4/42 (9.5)	20/52 (38.5)	14/52 (26.9)	6/52 (11.5)	19/45 (42.2)	15/45 (33.3)	4/45 (8.9)	34/97 (35.1)	25/97 (25.8)	9/97 (9.3)
* p* value	*0.800*	*0.763*		*0.820*	*0.941*		*0.729*	*0.445*		*0.244*	*0.360*	
mild	6/19 (31.6)	5/19 (26.3)	1/19 (5.3)	6/20 (30.0)	4/20 (20.0)	2/20 (10.0)	7/19 (36.8)	6/19 (31.6)	1/19 (5.3)	12/35 (34.3)	9/35 (25.7)	3/35 (8.6)
Moderate/ evere	10/23 (43.5)	7/23 (30.4)	3/23 (13.0)	14/32 (43.8)	10/32 (31.3)	4/32 (12.5)	12/26 (46.2)	9/26 (34.6)	3/26 (11.5)	22/62 (35.5)	16/62 (25.8)	6/62 (9.7)
* p* value	*0.712*	*0.855*		*0.600*	*0.886*		*0.772*	*0.709*		*0.504*	*0.723*	
*Length*												
Adequate Growth	111/276 (40.2)	77/275 (28.0)	33/275 (12.0)	110/275 (40.0)	76/274 (27.7)	33/274 (12.0)	110/278 (39.6)	77/277 (27.8)	32/277 (11.6)	106/260 (40.8)	73/259 (28.2)	32/259 (12.4)
Faltering Growth	6/23 (26.1)	2/23 (8.7)	4/23 (17.4)	7/24 (29.2)	3/24 (12.5)	4/24 (16.7)	7/21 (33.3)	2/21 (9.5)	5/21 (23.8)	11/39 (28.2)	6/39 (15.4)	5/39 (12.8)
* p* value	*0.182*	*0.125*		*0.297*	*0.257*		*0.572*	*0.085*		*0.134*	*0.229*	
*Head circumference*												
Adequate Growth	114/283 (40.3)	77/282 (27.3)	36/282 (12.8)	112/281 (39.9)	75/280 (26.8)	36/280 (12.9)	114/286 (39.9)	77/285 (27.0)	36/285 (12.6)	112/278 (40.3)	76/277 (27.4)	35/277 (12.6)
Faltering Growth	5/16 (31.3)	3/16 (18.8)	2/16 (12.5)	7/18 (38.9)	5/18 (27.8)	2/18 (11.1)	5/13 (38.5)	3/13 (23.1)	2/13 (15.4)	7/21 (33.3)	4/21 (19.0)	3/21 (14.3)
* p* value	*0.473*	*0.738*		*0.935*	*0.976*		*0.920*	*0.928*		*0.530*	*0.705*	

Chi-square/Fisher’s exact tests were performed. Data presented as *n* (%). *P* values under “any” are for comparisons between growth status (adequate or faltering growth) and any neurodisability while *p* values under “mild“ are for comparisons between growth status and severity of neurodisability.

Bold values indicate statistical significance *p* < 0.05.

Changes in head circumference *z*-scores between birth and 36 weeks’ PMA and also between 4 weeks and 36 weeks’ PMA for all four growth references were weakly positively correlated with cognitive, language and motor scores at 2 years (Supplementary Table 2). Faltering weight, length and head growth were more strongly associated with motor delay than cognitive or language delay (OR 1.9−4.7 vs OR 1.6−2.1). Faltering head growth between 4 and 36 weeks’ PMA by the fetal references was associated with the highest odds of motor delay compared to INTERGROWTH-21st (OR 3.4−4.7 vs 2.3). Generally, faltering head growth was more strongly associated with developmental delay than other measurements (OR 1.7−4.7 vs OR 1.6−2.4). Similarly, faltering growth between 4- 36 weeks’ PMA was more strongly associated with developmental delay than other time periods (OR 1.6−4.7 vs OR 1.7−2.9) (Table 5).

**Table 5:** Odds of developmental delay at 2 years’ corrected age in infants with faltering growth, classified using Fenton 2013, UK-WHO, Olsen 2010 and INTERGROWTH-21st.

Growth measure	Bayley-III composite scores <85	Fenton OR (95% CI)	UK-WHO OR (95% CI)	Olsen OR (95% CI)	IG-21st OR (95% CI)
Birth to 4 weeks					
Weight					
	Cognitive	1.2 (0.6−2.2)	1.3 (0.6−2.4)	1.1 (0.6−2.1)	0.4 (0.1−3.4)
	Language	1.3 (0.8−2.2)	1.1 (0.6−1.9)	1.1 (0.7−1.8)	0.4 (0.1−1.7)
	Motor	1.3 (0.7−2.5)	1.3 (0.7−2.5)	1.3 (0.7−2.3)	0.4 (0.1−3.2)
Length					
	Cognitive	1.1 (0.6−2.0)	0.7 (0.3−1.7)	0.9 (0.5−1.7)	1.6 (0.7−3.5)
	Language	1.3 (0.8−2.1)	1.1 (0.6−2.1)	1.2 (0.8−2.0)	1.4 (0.7−2.6)
	Motor	1.3 (0.7−2.4)	0.8 (0.3−2.1)	1.1 (0.6−2.0)	1.3 (0.6−2.9)
Head circumference					
	Cognitive	1.0 (0.6−1.9)	1.0 (0.54−1.81)	0.8 (0.44−1.46)	1.0 (0.56−1.86)
	Language	1.8^a^ (1.1−3.0)	1.5 (0.9−2.5)	1.3 (0.8−2.2)	1.5 (0.9−2.4)
	Motor	1.3 (0.72−2.45)	1.4 (0.8−2.6)	1.2 (0.6−2.1)	1.6 (0.9−3.0)
Birth to 36 weeks					
Weight					
	Cognitive	1.0 (0.5−1.9)	1.3 (0.6−2.5)	1.4 (0.8−2.7)	1.6 (0.7−3.7)
	Language	0.8 (0.5−1.3)	1.0 (0.6−1.8)	1.0 (0.6−1.7)	1.1 (0.5−2.4)
	Motor	1.1 (0.6−2.1)	1.2 (0.6−2.4)	1.5 (0.8−2.7)	1.8 (0.8−1.1)
Length					
	Cognitive	1.0 (0.5−1.9)	0.8 (0.4−1.7)	0.9 (0.5−1.6)	1.6 (0.9−3.0)
	Language	1.6 (1.0−2.7)	1.2 (0.7−2.3)	1.7 (1.0−2.8)	2.1^a^ (1.3−3.5)
	Motor	1.9 (1.0−3.7)	1.0 (0.5−2.2)	1.6 (0.8−3.2)	1.9^a^ (1.0−3.6)
Head circumference					
	Cognitive	1.1 (0.6−2.0)	1.1 (0.6−2.2)	1.1 (0.6−2.0)	1.7 (0.9−3.1)
	Language	1.4 (0.9−2.3)	1.2 (0.7−2.1)	1.2 (0.8−2.0)	1.9^a^ (1.2−3.1)
	Motor	2.1^a^ (1.2−3.8)	2.6^a^ (1.4−4.7)	2.3^a^ (1.3−4.2)	2.9^a^ (1.6−5.4)
4 weeks to 36 weeks					
Weight					
	Cognitive	1.4 (0.6−3.1)	0.9 (0.3−2.6)	1.2 (0.5−3.0)	1.1 (0.6−2.5)
	Language	0.9 (0.4−1.8)	0.7 (0.3−1.8)	0.8 (0.4−1.8)	1.4 (0.8−2.4)
	Motor	1.8 (0.8−3.8)	1.4 (0.5−3.7)	2.0 (0.9−4.7)	2.3^a^ (1.2−4.3)
Length					
	Cognitive	0.8 (0.4−1.7)	1.1 (0.5−2.3)	0.9 (0.4−1.8)	1.2 (0.7−2.3)
	Language	1.5 (0.9−2.6)	1.2 (0.7−2.2)	1.6 (0.9−2.7)	1.6^a^ (1.0−2.7)
	Motor	2.4^a^ (1.3−4.5)	2.0^a^ (1.0−3.9)	2.1^a^ (1.1−4.0)	2.2^a^ (1.2−4.1)
Head circumference					
	Cognitive	2.1 (0.7−6.1)	2.7 (0.9−8.3)	2.1 (0.7−6.1)	1.5 (0.6−3.4)
	Language	2.0 (0.7−5.3)	2.2 (0.8−6.6)	2.3 (0.9−5.9)	2.1^a^ (1.0−4.3)
	Motor	3.4^a^ (1.3−9.2)	4.7^a^ (1.6−13.7)	3.4^a^ (1.3−9.2)	2.3^a^ (1.1−5.1)
36 weeks to 2 years					
Weight					
	Cognitive	0.7 (0.3−1.8)	0.8 (0.3−1.9)	0.8 (0.3−2.0)	0.7 (0.4−1.5)
	Language	1.0 (0.5−2.0)	1.1 (0.6−2.0)	1.1 (0.6−2.2)	0.8 (0.5−1.4)
	Motor	1.0 (0.4−2.5)	1.3 (0.6−2.8)	1.6 (0.7−3.5)	1.0 (0.5−1.9)
Length					
	Cognitive	1.1 (0.4−3.4)	1.0 (0.3−3.2)	1.7 (0.6−4.9)	0.9 (0.4−2.4)
	Language	0.6 (0.2−1.6)	0.7 (0.3−1.9)	0.9 (0.3−2.3)	0.6 (0.3−1.4)
	Motor	1.4 (0.5−4.0)	1.7 (0.7−4.6)	2.1 (0.8−5.7)	1.1 (0.5−2.6)
Head circumference					
	Cognitive	0.3 (0.0−2.6)	0.3 (0.0−2.3)	0.4 (0.1−3.5)	0.5 (0.1−2.4)
	Language	0.5 (0.1−1.9)	0.6 (0.2−2.0)	0.7 (0.2−2.6)	0.5 (0.2−1.6)
	Motor	0.7 (0.2−3.3)	1.0 (0.3−3.7)	1.0 (0.2−4.52)	1.2 (0.4−3.8)

*OR* odds ratio, 95% *CI* confidence interval, *IG-21st* INTERGROWTH-21st.

^a^Odds ratio significant at *p* < 0.05.

The AUC for prediction of developmental delay were all <0.63. Faltering growth from birth to 4 weeks and birth to 36 weeks’ PMA as assessed using INTERGROWTH-21st was more specific but less sensitive for predicting developmental delay than fetal references. From birth to 36 weeks’ PMA, faltering head growth using INTERGROWTH-21st but not the fetal references predicted motor delay at 2 years (AUC = 0.62, 95%CI 0.54−0.71, *p* = 0.007) and faltering length growth also using INTERGROWTH-21st predicted language delay (AUC = 0.60, 95%CI 0.53–0.67, *p* = 0.007). Faltering length growth from 4 to 36 weeks’ PMA using INTERGROWTH-21st (AUC = 0.60, 95%CI 0.51–0.68, *p* = 0.034) and Fenton (AUC = 0.60, 95%CI 0.51–0.69, *p* = 0.029) predicted motor delay. Faltering in any growth measure did not significantly predict cognitive delay using any of the growth references. (Table 6).

**Table 6:** Faltering growth categorized by Fenton 2013, UK-WHO, Olsen 2010 and INTERGROWTH-21st as a predictor of developmental delay at 2 years’ corrected age.

Growth Measure	Bayley-III composite scores <85	Fenton			UK-WHO			Olsen			IG-21st		
		Sensitivity	Specificity	AUC (95% CI)	Sensitivity	Specificity	AUC (95% CI)	Sensitivity	Specificity	AUC (95% CI)	Sensitivity	Specificity	AUC (95% CI)
Birth to 4 weeks													
Weight													
	Cognitive	0.33	0.70	0.54 (0.44–0.63)	0.29	0.75	0.54 (0.45–0.63)	0.39	0.64	0.53 (0.44–0.63)	0.02	0.96	0.49 (0.40–0.58)
	Language	0.35	0.71	0.53 (0.46–0.60)	0.27	0.75	0.51 (0.44–0.58)	0.39	0.64	0.52 (0.45–0.59)	0.02	0.95	0.49 (0.42–0.56)
	Motor	0.36	0.70	0.54 (0.45–0.63)	0.30	0.75	0.53 (0.44–0.63)	0.42	0.64	0.54 (0.45–0.63)	0.02	0.96	0.49 (0.40–0.57)
Length													
	Cognitive	0.51	0.51	0.49 (0.39–0.60)	0.20	0.74	0.47 (0.37–0.57)	0.49	0.49	0.46 (0.36–0.57)	0.20	0.86	0.53 (0.44–0.62)
	Language	0.53	0.53	0.53 (0.45–0.62)	0.27	0.75	0.51 (0.43–0.59)	0.54	0.51	0.53 (0.44–0.61)	0.18	0.86	0.52 (0.45–0.59)
	Motor	0.55	0.52	0.53 (0.42–0.64)	0.23	0.74	0.49 (0.38–0.59)	0.53	0.49	0.51 (0.41–0.62)	0.18	0.86	0.52 (0.43–0.61)
Head circumference													
	Cognitive	0.59	0.42	0.49 (0.40–0.58)	0.55	0.45	0.50 (0.41–0.59)	0.55	0.40	0.47 (0.38–0.56)	0.53	0.49	0.50 (0.41–0.59)
	Language	0.68	0.46	0.56 (0.49–0.63)	0.62	0.48	0.55 (0.48–0.62)	0.64	0.43	0.53 (0.46–0.60)	0.59	0.51	0.55 (0.48– 0.62)
	Motor	0.64	0.43	0.54 (0.45–0.63)	0.62	0.46	0.54 (0.45–0.63)	0.62	0.41	0.52 (0.42–0.61)	0.62	0.49	0.56 (0.47–0.64)
Birth to 36 weeks													
Weight													
	Cognitive	0.35	0.66	0.49 (0.40–0.58)	0.26	0.79	0.51 (0.42–0.60)	0.39	0.69	0.54 (0.45–0.64)	0.16	0.90	0.51 (0.42–0.60)
	Language	0.32	0.64	0.47 (0.40–0.54)	0.22	0.79	0.50 (0.42–0.57)	0.32	0.68	0.50 (0.43–0.57)	0.12	0.89	0.50 (0.43–0.57)
	Motor	0.38	0.65	0.49 (0.40–0.59)	0.25	0.78	0.48 (0.39–0.57)	0.40	0.69	0.54 (0.44–0.63)	0.17	0.90	0.52 (0.43–0.61)
Length													
	Cognitive	0.60	0.40	0.47 (0.37–0.57)	0.31	0.64	0.47 (0.37–0.58)	0.60	0.36	0.45 (0.35–0.56)	0.54	0.57	0.56 (0.47–0.65)
	Language	0.68	0.44	0.52 (0.43–0.60)	0.39	0.66	0.53 (0.44–0.61)	0.71	0.41	0.52 (0.43–0.60)	0.57	0.62	0.60^a^ (0.53–0.67)
	Motor	0.72	0.42	0.53 (0.42–0.64)	0.36	0.64	0.50 (0.39–0.61)	0.72	0.39	0.51 (0.40–0.62)	0.58	0.58	0.59 (0.50–0.67)
Head circumference													
	Cognitive	0.45	0.57	0.51 (0.42–0.60)	0.33	0.70	0.51 (0.42–0.60)	0.47	0.55	0.51 (0.42–0.60)	0.49	0.64	0.55 (0.46–0.64)
	Language	0.49	0.60	0.54 (0.47–0.61)	0.33	0.71	0.51 (0.44–0.58)	0.49	0.57	0.53 (0.46–0.60)	0.49	0.67	0.57 (0.50–064)
	Motor	0.59	0.60	0.58 (0.49–0.67)	0.49	0.73	0.58 (0.49–0.67)	0.62	0.58	0.59 (0.50–0.68)	0.60	0.66	0.62^a^ (0.54–0.71)
4 weeks to 36 weeks													
Weight													
	Cognitive	0.18	0.87	0.51 (0.42–0.60)	0.08	0.91	0.49 (0.40–0.57)	0.12	0.90	0.50 (0.41–0.59)	0.28	0.75	0.51 (0.42–0.60)
	Language	0.13	0.86	0.49 (0.42–0.56)	0.07	0.91	0.48 (0.42–0.55)	0.09	0.89	0.49 (0.42–0.56)	0.30	0.77	0.53 (0.46–0.60)
	Motor	0.21	0.87	0.53 (0.44–0.62)	0.11	0.92	0.51 (0.42–0.59)	0.17	0.91	0.53 (0.44–0.62)	0.40	0.78	0.58 (0.49–0.67)
Length													
	Cognitive	0.25	0.72	0.49 (0.40–0.57)	0.22	0.79	0.51 (0.42–0.60)	0.25	0.74	0.49 (0.40–0.58)	0.49	0.56	0.52 (0.43–0.61)
	Language	0.33	0.76	0.54 (0.47–0.61)	0.23	0.81	0.52 (0.45–0.59)	0.32	0.77	0.54 (0.47–0.61)	0.53	0.60	0.55 (0.48–0.62)
	Motor	0.43	0.76	0.60^a^ (0.51–0.69)	0.31	0.81	0.56 (0.47–0.65)	0.39	0.77	0.58 (0.49–0.67)	0.61	0.59	0.60^a^ (0.51–0.68)
Head circumference													
	Cognitive	0.10	0.95	0.52 (0.43–0.60)	0.10	0.96	0.52 (0.43–0.61)	0.10	0.95	0.52 (0.43–0.61)	0.16	0.89	0.51 (0.42–0.60)
	Language	0.08	0.96	0.52 (0.44–0.59)	0.07	0.97	0.52 (0.44–0.59)	0.09	0.96	0.52 (0.44–0.59)	0.17	0.91	0.54 (0.47–0.61)
	Motor	0.13	0.96	0.54 (0.45–0.63)	0.13	0.97	0.54 (0.45–0.63)	0.13	0.96	0.54 (0.45–0.63)	0.21	0.90	0.54 (0.45–0.63)
36 weeks to 2 years													
Weight													
	Cognitive	0.10	0.86	0.48 (0.40–0.57)	0.14	0.83	0.49 (0.40–0.57)	0.12	0.86	0.49 (0.40–0.57)	0.26	0.68	0.47 (0.38–0.55)
	Language	0.13	0.87	0.50 (0.43–0.57)	0.17	0.84	0.51 (0.44–0.57)	0.15	0.86	0.51 (0.44–0.58)	0.28	0.68	0.48 (0.41–0.55)
	Motor	0.14	0.87	0.50 (0.42–0.59)	0.19	0.84	0.52 (0.43–0.61)	0.19	0.87	0.53 (0.44–0.62)	0.31	0.69	0.50 (0.42–0.59)
Length													
	Cognitive	0.08	0.92	0.50 (0.41–0.59)	0.08	0.92	0.50 (0.41–0.59)	0.10	0.94	0.52 (0.43–0.61)	0.13	0.87	0.50 (0.41–0.59)
	Language	0.05	0.91	0.48 (0.41–0.55)	0.07	0.91	0.49 (0.42–0.56)	0.07	0.93	0.50 (0.43–0.57)	0.10	0.85	0.48 (0.41–0.55)
	Motor	0.10	0.93	0.51 (0.42–0.60)	0.12	0.93	0.52 (0.43–0.61)	0.12	0.94	0.53 (0.44–0.62)	0.14	0.87	0.51 (0.42–0.59)
Head circumference													
	Cognitive	0.02	0.94	0.48 (0.40–0.57)	0.02	0.94	0.48 (0.39–0.56)	0.02	0.96	0.49 (0.40–0.57)	0.04	0.93	0.48 (0.40–0.57)
	Language	0.03	0.94	0.49 (0.42–0.56)	0.04	0.94	0.49 (0.42–0.56)	0.03	0.96	0.49 (0.42–0.56)	0.04	0.92	0.48 (0.41–0.55)
	Motor	0.04	0.95	0.49 (0.41–0.58)	0.06	0.94	0.50 (0.41–0.59)	0.04	0.96	0.50 (0.41–0.59)	0.08	0.94	0.51 (0.42–0.59)

*AUC* area under receiver operating characteristic curve, *IG-21st* INTERGROWTH-21st.

^a^*p* < 0.05.

Sensitivity analysis excluding the 20 infants born at 23 weeks whose *z*-scores for INTERGROWTH-21st were calculated by extrapolation did not change our findings (Supplementary Tables 3–7).

Sensitivity analysis excluding SGA infants also did not change any of our findings about the relationships between faltering growth and developmental delay at 2 years (Supplementary Tables 8 and 9).

## Discussion

We examined the differences in classification of postnatal growth using fetal references (Fenton, UK- WHO and Olsen) and a healthy preterm growth standard (INTERGROWTH-21st) and their association with neurodevelopment at 2 years’ CA. Although faltering early growth was poorly predictive of developmental delay using any of the growth charts, our results suggest that infants with faltering postnatal growth classified by INTERGROWTH-21st were at highest risk of developmental delay compared to those classified using fetal references, and that faltering growth in head circumference and length, particularly from 4 to 36 weeks’ PMA, were most strongly associated with developmental delay at 2 years.

We found that there was high agreement between the fetal references in the classification of faltering growth. In general, infants categorized as having faltering growth by Fenton, were also categorized as having faltering growth by UK- WHO and Olsen. However, there was less agreement between INTERGROWTH-21st, the healthy preterm standard, and the fetal references. These findings are in agreement with other reports of higher agreement in faltering growth classification between fetal references than between INTERGROWTH-21st and fetal references^24^. This is likely attributable to the differences in methods used to develop the different growth curves. The fetal references were developed from cross-sectional birth data of infants born preterm^8,15^ whereas the INTERGROWTH-21st preterm growth curves were developed from longitudinal growth measures of fetuses who were subsequently born preterm.^14^ Thus, the fetal growth curves are similar to each other but different from the preterm growth standards.

Olsen charts identified the highest prevalence of faltering growth. However, faltering growth using INTERGROWTH-21st was more strongly associated with developmental delay than faltering growth using the fetal references, although with some overlap of CI. The fetal charts, unlike the INTERGROWTH-21st, are based on in utero growth that does not account for the early physiological weight loss and slightly longer time required by preterm infants to regain birthweight, thus preterm infants are more likely to be classified as having faltering growth using these references than by the preterm postnatal growth standards of INTERGROWTH-21st, particularly within the first four postnatal weeks. Therefore INTERGROWTH-21st chart detects more severe faltering growth, possibly accounting for the observed higher association with developmental delay using the preterm growth standard. We also found that from 36 weeks’ PMA to 2 years, faltering growth using any of the growth charts was not associated with increased risk of developmental delay. This suggests there is no advantage in using any particular one of these growth charts at 36 weeks’ PMA (approximately NICU discharge) for identifying infants at risk of developmental delay.

An inherent limitation of the INTERGROWTH-21st healthy preterm growth standards is the inclusion of very few infants born extremely or very preterm. Thus, for our study, we could not directly calculate birth *z*-scores of weight, length and head circumference from the INTERGROWTH-21st Very Preterm Size at Birth References for 20 (6.1%) participants born at 23 weeks and had to extrapolate the *z*-scores from later gestations. However, sensitivity analysis excluding these extrapolated values did not change our findings.

We found that faltering growth using any of the four growth references was not a good predictor of developmental delay at 2 years’ CA, with AUC all <0.63. This finding is in agreement with and extends the findings of a previous study of infants born <33 weeks’ gestation that growth measured using Fenton, Olsen and INTERGROWTH-21st were not strong predictors of developmental delay at 18 months and 7 years, and that the AUC were all <0.69.^24^ A retrospective study of infants born <33 weeks’ gestation that assessed faltering growth using Fenton and INTERGROWTH-21st as predictors of developmental delay at 12 and 24 months also reported that the AUC were all <0.66.^25^ Although being born SGA is associated with poorer neurodevelopmental outcomes,^26,27^ adjusting for SGA did not improve the predictive ability of faltering weight growth for poor neurodevelopmental outcomes in a previous study.^25^ Our sensitivity analysis excluding SGA infants also did not change our findings, with the odds of developmental delay largely unchanged and all AUC < 0.63. Many factors other than growth, including illness, infection and brain injury, contribute to neurodevelopmental outcomes, and these factors may also contribute to faltering growth in affected infants.^28,29^ Thus, faltering growth may serve as a marker for identifying infants at risk, rather than being a good predictor of developmental delay by itself. Nevertheless, prevention of faltering growth in NICU remains a key goal, as faltering growth is associated with later developmental delay and could also exacerbate other morbidities.

Many authors have reported that faltering growth during NICU stay, typically up to term-equivalent age, is related to adverse neurodevelopmental outcomes,^23,24,30,31^ but there is less certainty about which period is most important. Others have noted the frequency of faltering growth in the first few postnatal weeks owing to early weight loss from postnatal diuresis and delayed initiation of nutrition support, among other reasons.^10,32,33^ Our findings suggest that this early faltering growth in the first 4 weeks is not strongly related to later developmental delay. Rather, it is continuing faltering growth from 4 to 36 weeks’ PMA, particularly if severe, that is associated with increased risk of later developmental delay. This may be consistent with findings of a recent study that faltering head growth calculated from the end of physiological weight loss (14–21 postnatal days) rather than from birth, to discharge, was associated with an increased risk of neurodevelopmental impairment at 24 months’ CA in very preterm infants (aOR = 3.94, 1.19–13.03 vs aOR = 0.69, 0.33–1.45).^34^ It remains to be seen whether a focus on improving growth in the NICU, particularly between 4 and 36 weeks’ PMA, may help attenuate risk of later developmental delay. It is also possible infants taking part in the ProVIDe trial of early nutrition received better early nutrition than may be the case in other centers, so the generalizability of this finding needs further assessment.

We found that faltering head growth was more strongly associated with developmental delay than faltering weight or length growth. Other studies similarly found that faltering head growth in infancy was associated with higher odds of developmental delay compared to faltering weight or length growth.^24,34,35^ Head growth is an indicator of brain growth, thus faltering head growth likely reflects poor brain growth,^36^ which is an independent predictor of faltering neurodevelopmental outcomes.^37^

Faltering growth in all growth measures was more strongly associated with motor delay than cognitive or language delay. This may be because at 2 years, motor skills are more easily assessed and delays more readily identified than delays in higher order cognitive or language skills,^38^ or that development of motor function is more affected by early nutrition. Nutritional supplements are reported to have a greater effect on motor outcomes than other aspects of development in infants (<3 years) who were born preterm or SGA.^39^ It remains to be seen whether this association persists at early school-age follow-up of this cohort. Other authors have reported among infants born extremely preterm, that neurodevelopmental outcomes in mid-childhood are poorly predicted by outcomes at 2 years.^38^

Our study has many strengths. Firstly, our study focuses on ELBW infants who are at the highest risk of poor nutritional and neurodevelopmental outcomes compared to other preterm and term-born children Second, anthropometric measurements were conducted prospectively according to standardized methods, and we were able to analyze longitudinal changes in growth over time as well as cross-sectional data. The cohort size was large and the follow-up at 2 years’ CA was very high (94%), so the results of this study are generalizable to the ProVIDe trial cohort and also to ELBW of similar characteristics to this RCT cohort.

There are also some limitations. Because infants enrolled in the ProVIDe Trial are likely to have been the most unwell and smallest for whom clinicians had chosen to insert a UAC, the generalizability of our study’s findings may be more limited for ELBW infants who are less sick, and sample size was limited by the size of the inception cohort. Although our cohort was of similar size to others reporting relationships between early growth and later development in preterm infants,^25,40,41^ validation of our findings with other and ideally larger cohorts of ELBW infants is warranted. It is also likely that as part of routine care, clinicians would have intervened to mitigate any detected faltering growth, thus potentially reducing the strength of any observed associations between faltering early growth and developmental delay. We also acknowledge that neurodevelopmental assessment at 2 years has limited predictive value for later childhood neurodevelopmental outcomes, and thus further follow-up of this cohort at school age is currently underway.

In summary, the highest prevalence of faltering growth was recorded using Olsen growth charts. Faltering head growth and faltering growth from 4 weeks to 36 weeks’ PMA were associated with developmental delay at 2 years, particularly motor delay. Although faltering growth detected with growth charts may help identify infants at risk, it is poorly predictive of developmental delay at 2 years’ CA.

### Supplementary information

Supplementary Tables

## Data Availability

The datasets used and/analyzed for this current study are available from the Liggins Institute Data Access Committee upon reasonable request.
